# Influenza virus infection mimicking an acute abdomen in a female adolescent

**DOI:** 10.1111/irv.12222

**Published:** 2013-12-26

**Authors:** Karina L Vivar, Timothy M Uyeki

**Affiliations:** a Department of Pediatrics, University of California San FranciscoSan Francisco, CA, USA; b Influenza Division, Centers for Disease Control and PreventionAtlanta, GA, USA

**Keywords:** Abdominal pain, cytokine storm, hypercytokinemia, influenza

## Abstract

We report an adolescent with respiratory symptoms admitted for clinical signs of an acute abdomen. The only diagnostic finding was influenza A viral RNA detected in an upper respiratory tract specimen. Influenza should be considered in the differential diagnosis of children with respiratory illness and abdominal pain during influenza season.

Seasonal influenza A or B virus infection generally causes self-limited, uncomplicated febrile upper respiratory tract illness. However, children may experience severe complications, such as pneumonia, encephalopathy, encephalitis, secondary invasive bacterial infection, myocarditis, myositis, rhabdomyolysis, hepatitis, and sudden death.^1^

A 17-year-old female adolescent presented to a hospital complaining of 2 days of diffuse abdominal pain. She also reported daily heavy vaginal bleeding and had a history of *Chlamydia trachomatis* genitourinary infection. Other acute symptoms included rhinorrhea, cough, feverishness, myalgias, and headache. Workup included pelvic exam, complete blood count, electrolytes, urinalysis, and chest Xray—all unremarkable. She received intravenous hydromorphone for analgesia and was discharged without a definitive diagnosis.

The next day, the patient presented with worsening abdominal pain, nausea, anorexia, and diarrhea. She was febrile, tachycardic, toxic-appearing, and mildly dehydrated. Abdominal exam was significant for hypo-active bowel sounds, exquisite tenderness to light palpation, worst in the right lower quadrant, with rebound tenderness and guarding, concerning for an acute abdomen requiring emergency surgical intervention. The patient was admitted for urgent evaluation of gastrointestinal, gynecologic, and genitourinary etiologies.

Laboratory results revealed mild leukopenia and lymphopenia, slightly elevated transaminases, and elevated partial thromboplastin time and D-dimer. Abdominal computed tomography, transvaginal ultrasound, gynecologic examination, and genitourinary workup were non-diagnostic. The only significant finding was influenza A viral RNA detected by RT-PCR in a nasopharyngeal specimen collected on the 3rd illness day. She was discharged after 2·5 days of supportive care, intravenous opiate analgesia, and hydration.

Gastrointestinal symptoms associated with influenza include anorexia, nausea, vomiting, and diarrhea, all of which are more common in children than in adults. Diarrhea is more frequent in younger children with influenza and with influenza B.^2^,^3^ Diarrhea was also common during the 2009 H1N1 pandemic. Severe abdominal pain associated with influenza may occur in school-aged children, and there are case reports of influenza mimicking an acute abdomen as in our patient.^4^,^5^ Elevation of transaminases may occur, and rare cases of hepatic failure have been reported.^6^ Reye's syndrome, often associated with salicylate exposure, and more commonly with influenza B, may result in fatal outcomes.^7^

The pathogenesis of gastrointestinal symptoms associated with influenza is unclear. Seasonal influenza A and B viral RNA has been detected in stool specimens from patients with respiratory symptoms and diarrhea.^8^–^10^ Additionally, highly pathogenic avian influenza A (H5N1) virus has been detected in feces and in intestinal mucosa.^11^ It has been hypothesized that influenza viruses may bind to alpha 2,3 sialic acid receptors in the human gastrointestinal tract, allowing entrance into and replication within gastrointestinal tract epithelial cells.^12^ However, neither seasonal influenza viral antigens nor human influenza virus receptors have been found within intestinal epithelial cells.^13^ Detection of seasonal influenza viral RNA in feces has been proposed to be a consequence of swallowed influenza viruses from the upper respiratory tract or remnants of infected submucosal intestinal antigen-presenting immune cells.^14^,^15^

Rather than extrapulmonary influenza virus infection, the pathogenesis of abdominal pain is more likely attributable to influenza virus infection of the upper respiratory tract triggering an exuberant host inflammatory response. In murine models and *in vitro* studies, human influenza viruses have been shown to elicit cytokine dysregulation, attracting inflammatory leukocytes, and inducing excessive cytokine levels. This process can result in diffuse capillary permeability, resulting in systemic shock and distal organ injury such as cerebral edema and potentially also intestinal damage.^16^

In summary, influenza should be considered in the differential diagnosis of children with febrile respiratory illness and severe abdominal pain during influenza season, after excluding those diagnoses that require urgent surgical intervention. Early empiric antiviral treatment and influenza testing are recommended if influenza is suspected, especially if the patient is hospitalized.
